# Supplementary material for: Tunable inverted gap in monolayer quasi-metallic MoS2 induced by strong charge-lattice coupling
Source: Nat Commun. 2017 Sep 7;8:486. doi: 10.1038/s41467-017-00640-2 (PMC5589873; doi:10.1038/s41467-017-00640-2)
Supplement: Supplementary file 2 — Supplementary Information [file 41467_2017_640_MOESM2_ESM.pdf]

Description of Supplementary Files

File Name: Supplementary Information
Description: Supplementary Figures, Supplementary Notes, Supplementary Methods and Supplementary References

File Name: Peer Review File

File Name: Supplementary Information

Description: Supplementary Figures, Supplementary Notes, Supplementary Methods and 12 Supplementary References

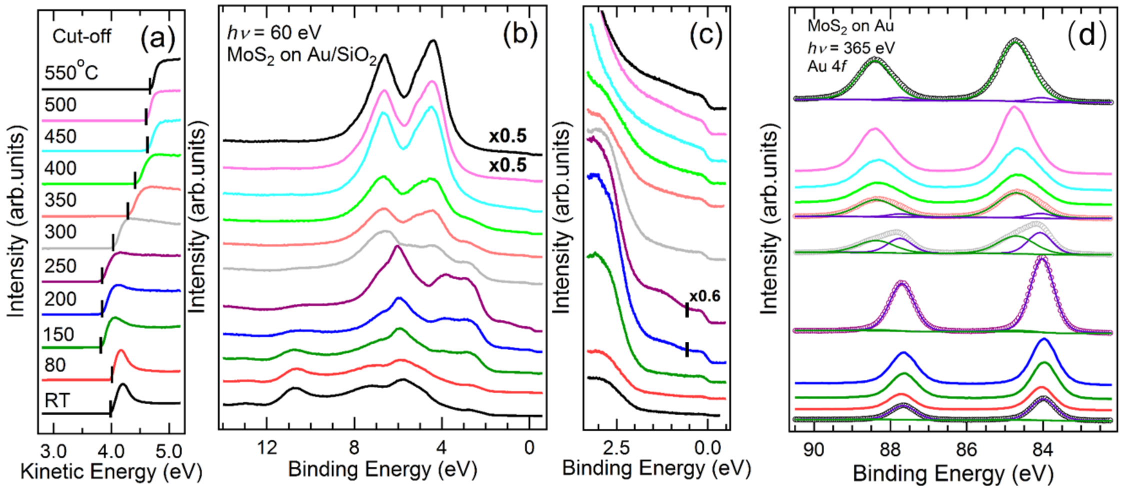

Supplementary Figure 1 | Annealing temperature-dependence of synchrotron based PES. a, work function, **b,** valence band, and **c,** narrow valence band for MoS_2 on Au film annealed at various temperatures. **d,** Au-4*f* core level spectra for MoS_2 on Au film annealed at various temperatures.

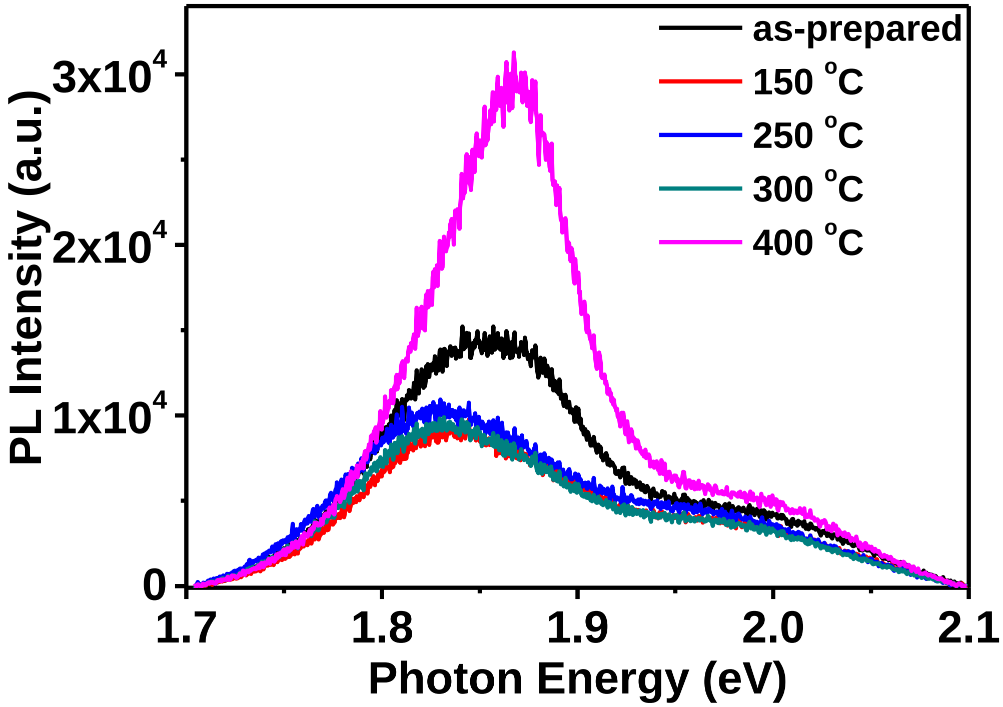

Supplementary Figure 2 | Annealing temperature-dependence of PL. The photoluminescence spectra of monolayer-MoS_2 on SiO_2/Si substrate in the device (Figure 2b in main text) annealed at respective temperatures.

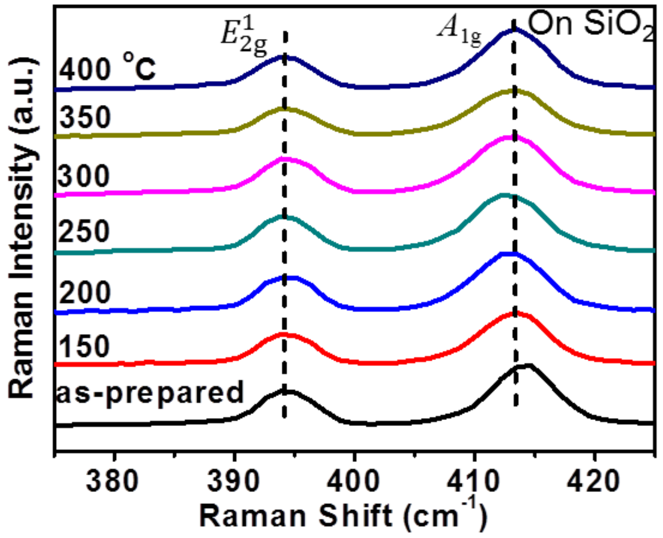

Supplementary Figure 3 | Annealing temperature-dependence of Raman spectroscopy. Raman spectra of MoS_2 film on SiO_2/Si substrate in the device (Figure 2b in main text) annealed at various temperatures.

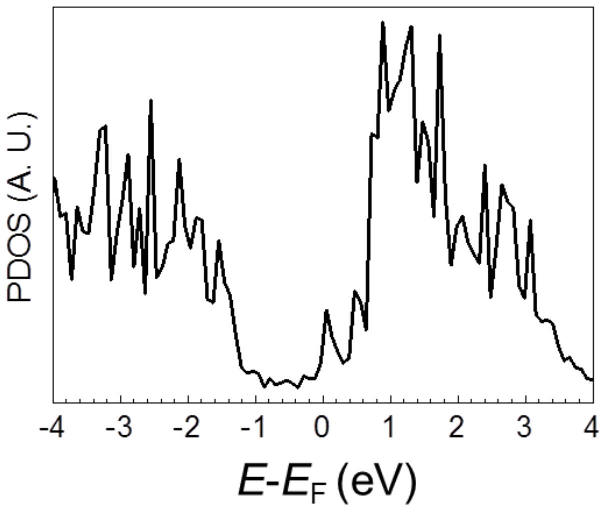

Supplementary Figure 4 | Calculated projected density of states (PDOS). Metallic projected density of states (PDOS) of MoS_2 with Au atom substitution on bottom Sulphur vacancy on Au(111) substrate. Similar PDOS for Au atom substitution top S vacancy.

Supplementary Note 1 – Work function and valence band. Another support for the phase-transition is the work function and valence band spectra which shown in Supplementary Figure 1a-c. The work function of MoS_2/Au, which is extracted from the secondary electron cutoff (cf. Supplementary Figure 1a) decreases slight upon annealing below 300°C, and then it increases generally, which is consistent with the transport results (including mobility and ON current). Seen from the narrow valence band spectra (Supplementary Figure 1c), a mid-peak appears at ~0.5eV when the sample was annealed at 200-250° Ç and it disappears at higher annealing temperatures. This mid-peak supports the inverted gap of the 1T' phase MoS_2 which is observed in the optical spectra (Figures 1f and g in main text).

Supplementary Note 2 - Photoluminescence Spectroscopy (PL) measurement. The PL spectra of the FET (Figure 2b in main text) after annealing at different temperatures are displayed in main text Figures 3d and e and Supplementary Figure 2. For MoS_2 on SiO_2/Si (Supplementary Figure 2), the emission intensity decreases slightly upon annealing at 150°C and rises again at 400°C. The slight intensity drop may be attributed to a better film-substrate contact after initial annealing. Whereas, the intensity increase after high-temperature annealing has been ascribed to the high quantum efficiency of excitons localized at the defect sites[1]. For as-prepared MoS_2/Au (main text Figure 3d), excitonic peaks *A* and *B* are located at 1.89 and 2.03eV respectively—in good agreement with report

on mechanically exfoliated monolayer samples.[2] After annealing at 200°C, the peaks are red-shifted and the intensity reduced–expected due to their partial metallic character.

Supplementary Note 3 - Metallic projected density of states (PDOS) calculation. The PDOS of MoS_2 are calculated with VASP package[3] employing the projector augmented wave (PAW) potential and within the GGA-PBE approximation[4], which is shown in Supplementary Figure 4. Van der Waals interactions were included using the PBE-D2 functional[5]. The MoS_2 supercell consists of a 5×5 primitive MoS_2 cell placed on a 6-layer Au(111) substrate that has been rotated to give an average in-plane strain of about 1.5%. The lattice parameters (a'=b') of the Au(111) supercell is consists of 6a+2b of primitive cell. Plane-wave cutoff energies are set at 336eV and larger than 15Å vacuum are used to avoid spurious interactions between neighboring slabs. A force convergence criterion of 0.05eV/Å is used. 2×2×1 gamma centered Monkhorst-Pack k grids for relaxation and 4×4×1 for PDOS are used.

Supplementary Note 4 – Au-4*f* PES. Supplementary Figure 1d shows the evolution of Au-4*f* core level spectra for the monolayer-MoS_2 on Au thin film on SiO_2/Si substrate before and after annealing at elevated temperature. The doublet associated with metallic gold is observed at 84eV. The spectral profile remains unchanged upon annealing below 300°C, and is broadened for further annealing, suggesting that a new feature is present. The metallic gold dominates below 300°C, which suggests that there is a weak chemical bond between Au and MoS_2. After then, new feature at ~84.7eV starts to dominate at 350°C, suggesting that the surface Au atoms are interacted with decomposed MoS_2. Combined

with the shift of lowest binding energy S-2p peak in main text Figure 3b, it is confirmed that Au-S bonds are formed at the near surface of Au film[6]. The S-Au bond is also shown in the S-2*p* PES spectra at high annealing temperature in main text Figure 3a. It demonstrated that the phase transition is not due to the chemical bond between Au and MoS_2. In main text Figures 3a and b, the Mo-3*d* signals vanish while the S-2*p* signals remains above 500°C. This indicates that the Mo atoms desorb from the Au surface and bulk diffusion takes place while S atoms form strong bonds with the Au surface.

Supplementary Methods

Spectroscopic ellipsometry measurement. We use a J. A. Woollam Co., Inc VASE and IR-VASE spectroscopic ellipsometer with photon energy of 0.4-4 eV and 0.07-0.17 eV to measure the ellipsometry parameters Ψ (the ratio between the amplitude of *p*-and *s*-polarized reflected light) and Δ (the phase difference between of *p*-and *s*-polarized reflected light). The dielectric function and absorption coefficient of MoS_2 monolayer was extracted from the parameters Ψ and Δ utilizing an air/MoS_2/Au (or Al_2O_3) multilayer model, where the monolayer-MoS_2 composed of an average homogeneous and uniform medium.[7, 8]

The Ψ and Δ defined as

$$\tan\Psi \exp(i\Delta) \equiv \frac{r_p}{r_s} \qquad (1)$$

where $r_{p(s)}$ is the reflectivity of *p*-(*s*-) polarized light. Using the Fresnel equations, the quantities could be defined as

$$r_p^{ij} = \frac{n_j \cos\theta_i - n_i \cos\theta_j}{n_j \cos\theta_i + n_i \cos\theta_j} \qquad (2)$$

and

$$r_s^{ij} = \frac{n_j \cos\theta_i - n_i \cos\theta_j}{n_j \cos\theta_i + n_i \cos\theta_j} \qquad (3)$$

Here *n* and *θ* represent the refractive index and angle of incident, respectively. The *i* and *j* represent the two materials. The complex dielectric function $\varepsilon(\omega) = \varepsilon_1(\omega) + i\varepsilon_2(\omega)$ of media can be obtained using

$$\sqrt{\varepsilon(\omega)} = n(\omega) \qquad (4)$$

where *ω* is the photon frequency.

The absorption coefficient of media is obtained as

$$\alpha = \frac{4\pi k}{\lambda} \qquad (5)$$

Here, k is the extinction coefficient (the imaginary part of the complex refractive index n) and λ is the light wavelength.

The reflectivity of MoS_2 film on substrate can be expressed as [9],

$$r_{multi} = \frac{r_{amb,MoS_2} + r_{MoS_2,sub}\exp\left(i2\delta_{MoS_2}\right)}{1 + r_{amb,MoS_2}\cdot r_{MoS_2,sub}\exp\left(i2\delta_{MoS_2}\right)} \qquad (6)$$

where

$$\delta_{MoS_2} = \frac{2\pi d_{MoS_2}}{\lambda}\sqrt{n_{MoS_2}^2 - n_{amb}^2\sin^2\theta} \qquad (7)$$

where the subscripts *multi* and *amb* represent monolayer-MoS_2 on Au (or Al_2O_3) substrate multilayer system and the ambient, respectively, while δ_{MoS2} is the change in light phase as it reflects from the MoS_2 film, and d_{MoS2} is the thickness of the MoS_2 film which is used 0.9nm including roughness for the average of thickness of monolayer MoS_2 grown by CVD[10]. For the refractive index of the substrate, Au and Al_2O_3, spectroscopic ellipsometry measurements were measured separately. One clean bulk Al_2O_3 and one clean Au film on SiO_2/Si were used.

Using the measured $\varepsilon(\omega)$ of substrate Au (and Al_2O_3), the $\varepsilon(\omega)$ of MoS_2 films can be extracted from Ψ and Δ through fitting [11] with Drude-Lorentz oscillators according to

$$\varepsilon(\omega) = \varepsilon_\infty + \sum_k \frac{\omega_{p,k}}{\omega_{0,k}^2 - \omega^2 - i\,\Gamma_k\omega} \qquad (8)$$

Here ε_∞ is the high frequency dielectric constant; $\omega_{p,k}$, $\omega_{0,k}$, and Γ_k are the plasma frequency, the transverse frequency (eigen-frequency), and the line width (scattering rate) of the k-th

oscillator, respectively. The absorption coefficient of MoS_2 monolayer is obtained through direct function inversion of Supplementary Equations 4 and 5.

Photoemission spectroscopy (PES) measurement. The synchrotron based PES data were taken in ultra-high vacuum chamber with a base pressure of 1×10^{-10} mbar at the SINS beamline of Singapore Synchrotron Light Source (SSLS)[12]. The measurements were performed immediately after the samples were cooling down to room temperature from high annealing temperatures.

The photon energy of 365eV was used to probe the Mo-3*d*, S-2*p* and Au-4*f* spectra (Figure 3 in main text, Supplementary Figure 1d and Supplementary Notes 4). The photon energy of 60eV was used to probe the valence band spectra (Supplementary Figures 1b,c and Supplementary Notes 1). The work function was measured using 60eV photon energy with a -7V applied bias (Supplementary Figure 1a and Supplementary Notes 1). All spectra were collected at normal emission using a VG Scienta R4000 analyzer and normalized by photon current.

The photon energy was calibrated using the Au $4f_{7/2}$ core level peak at 84.0eV of a sputter-cleaned gold foil in electrical contact with the sample. The binding energy is referred to the Fermi level of gold foil. The least-squares peak fit analysis were performed by using Voigt photoemission profiles with constant Lorentzian (15%) and Gaussian (85%) line shape. For S-2*p* and Mo-3*d* spectra fitting in Figure 3 in main text, splitting difference of

~1.18eV with branching ratio of 2 ($2p_{3/2}$) : 1 ($2p_{1/2}$) and ~3.15eV with branching ratio of 3 ($3d_{5/2}$) : 2 ($3d_{3/2}$) were used respectively.

Supplementary References

1. Nan H, *et al.* Strong Photoluminescence Enhancement of MoS2 through Defect Engineering and Oxygen Bonding. *ACS Nano* **8**, 5738-5745 (2014).

2. Mak KF, Lee C, Hone J, Shan J, Heinz TF. Atomically Thin MoS2: A New Direct-Gap Semiconductor. *Phys. Rev. Lett.* **105**, 136805 (2010).

3. Kresse G, Furthmüller J. Efficient iterative schemes for ab initio total-energy calculations using a plane-wave basis set. *Phys. Rev. B* **54**, 11169-11186 (1996).

4. Perdew JP, Burke K, Ernzerhof M. Generalized Gradient Approximation Made Simple. *Phys. Rev. Lett.* **77**, 3865-3868 (1996).

5. Grimme S. Semiempirical GGA-type density functional constructed with a long-range dispersion correction. *J. Comput. Chem.* **27**, 1787-1799 (2006).

6. Quintiliani M, *et al.* Network assembly of gold nanoparticles linked through fluorenyl dithiol bridges. *J. Mater. Chem. C* **2**, 2517-2527 (2014).

7. Yin X, *et al.* Unraveling how electronic and spin structures control macroscopic properties of manganite ultra-thin films. *NPG Asia Mater.* **7**, e196 (2015).

8. Yin X, *et al.* Coexistence of Midgap Antiferromagnetic and Mott States in Undoped, Hole- and Electron-Doped Ambipolar Cuprates. *Phys. Rev. Lett.* **116**, 197002 (2016).

9. Harbecke B. Coherent and Incoherent Reflection and Transmission of Multilayer Structures. *Appl. Phys. B-Photo* **39**, 165-170 (1986).

10. Liu H-L, Shen C-C, Su S-H, Hsu C-L, Li M-Y, Li L-J. Optical properties of monolayer transition metal dichalcogenides probed by spectroscopic ellipsometry. *Appl. Phys. Lett.* **105**, 201905 (2014).

11. Kuzmenko A. Kramers–Kronig constrained variational analysis of optical spectra. *Rev. Sci. Instrum.* **76**, 083108 (2005).

12. Yu X, *et al.* New soft X-ray facility SINS for surface and nanoscale science at SSLS. *J. Electron Spectrosc. Relat. Phenom.* **144–147**, 1031-1034 (2005).
